# Case Report: Transcatheter Aortic Valve Replacement in a Patient With Severe Aortic Stenosis, Left Ventricular Dysfunction, and an Anomalous Left Circumflex Artery

**DOI:** 10.3389/fcvm.2021.721363

**Published:** 2021-08-18

**Authors:** Badreyah Aldauig, Mohammed El-Sabbah, Mirvat Alasnag

**Affiliations:** Cardiac Center, King Fahd Armed Forces Hospital, Jeddah, Saudi Arabia

**Keywords:** cardiac computed tomographic, transcatheter aorta valve replacement, anomalous coronary arteries, echocardiography, cardiac catheterization

## Abstract

The role of cardiac computed tomography in the evaluation of patients for transcatheter aortic valve implantation is well-established. However, its role in the evaluation of anomalous vessels in the pre-procedure planning, intra-procedural fusion imaging and post-procedure assessment of vessel patency is not yet defined. This case report illustrates the utility of cardiac CT throughout the management of complex structural interventions. Here, we describe an anomalous left coronary artery where the course of the anomalous vessel and its proximity to the aortic valve annulus is defined allowing the selection of the most appropriate balloon expandable valve with a planned deployment. Upon follow up, patency of this anomalous vessel is ascertained using CT as well as the transcatheter valve function and leaflet thickening.

## Introduction

Transcatheter aortic valve replacement (TAVR) is one of the fastest-growing procedures that have become a mainstream option for inoperable, high-, intermediate-, and even low-surgical-risk patients with severe aortic stenosis (1). Current data confirm immediate procedural results and long-term outcomes that are comparable to surgical valve replacement.

The currently available TAVR platforms are balloon and self-expanding valves, such as the balloon-expandable Edwards SAPIEN (Edwards Lifesciences, Irvine, CA, USA) and the self-expanding CoreValve® (Medtronic, Minneapolis, MN, USA) (1, 2). The limitation of TAVR technology is related to the incidence of complications such as paravalvular regurgitation, valve malposition, valve embolization, stroke, conduction disturbances, and coronary obstruction. Pre-procedure planning and imaging help predict and prevent such potential complications (2).

Cardiac computed tomography (CCT) is an important tool in the pre-procedural evaluation whereby the aortic valve morphology, annular area, annulus to coronary height, sino-tubular junction diameter, coronary anatomy, left ventricular function, and peripheral vasculature are assessed (3–5). In this report, we describe a patient with an anomalous coronary artery who underwent evaluation by CCT before and after the valve replacement to evaluate the anomalous left circumflex artery.

## Presentation

An 82-year-old female patient whose cardiovascular risk factors included uncontrolled hypertension and hyperlipidemia presented with a 3-month history of progressive dyspnea class II that worsened over the last 2 weeks. Her medications included perindopril 5 mg OD and atorvastatin 40 mg OD. Over the last 2 weeks, she noted multiple episodes of syncope. She did not describe any palpitations, chest pain, or intermittent claudication.

## Examination

On physical examination at admission, the patient's temperature was 36.3°C, pulse 85 bpm, respiration rate 19/min, and blood pressure (BP) 110/80 mmHg without orthostatic changes. The lungs displayed bilateral clear breathing sounds. There was a slow rising peripheral pulse associated with a delayed sustained peak. Auscultation revealed a grade 4/6 crescendo–decrescendo midsystolic ejection murmur heard best at the right upper sternal border radiating to the neck and carotid arteries. No diastolic murmur was heard. The apex beat was slightly displaced laterally at the left side, which suggests the evidence of left ventricular hypertrophy (LVH). No lower-extremity edema was detected.

## Investigations

The initial hematologic and biochemical investigations were within normal with a hemoglobin of 14.7 gm/dl, white blood cells 6,000/μl, creatinine 1.2 mg/dl, potassium 3.7 mg/L, sodium 140 mg/L, and alanine aminotransferase 25 U/L. The chest x-ray demonstrated a prominent right mediastinal border occupied by the ascending aorta. There was an enlarged aortic knob. An echocardiogram revealed a trileaflet aortic valve (AV) that was severely stenotic and calcified with a maximum gradient of 100 mmHg, a mean gradient of 60 mmHg, an AV annulus diameter of 19 mm, an impaired left ventricular systolic function with an ejection fraction (EF) of 35%, and mildly dilated left ventricle with mild LVH (Supplementary Figure 1). The transmitral spectral Doppler flow pattern is suggestive of impaired LV relaxation without regional wall motion abnormalities noted.

**Figure 1:**
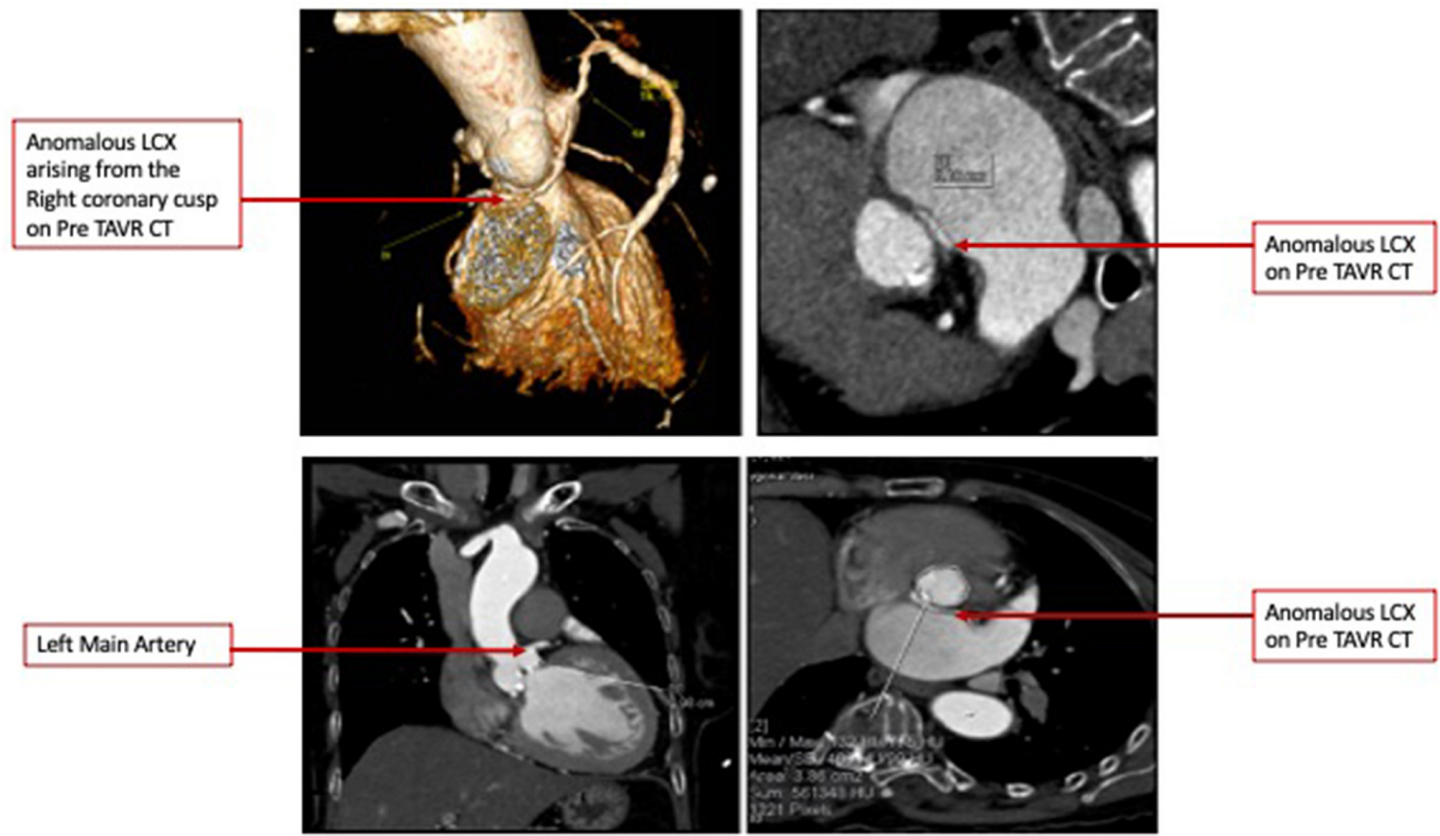
**(A)** Pre-TAVR Cardiac Computed Tomography 3D volume rendered images demonstrating an anomalous Left Circumflex Artery and Minimal Intensity Projections denoting a course between the Left Atrium and Aorta. **(B)** Pre-TAVR Cardiac Computed Tomography images demonstrating an Aortic valve annulus of 3.86 cm 2 and Annulus to Left Main distance of 10 mm.

CCT confirmed severe AS and revealed an anomalous LCX arising from the right coronary cusp, having a small caliber with diffuse disease (Figure 1A) and coursing between the aorta and left atrium. The right coronary artery was dominant with diffuse irregularities, especially distally. The AV annulus to the left main height was 0.98 cm, and the AV annulus area is 3.98 cm^2^ (Figure 1B).

## Management (Medical/Interventional)

**Figure 2:**
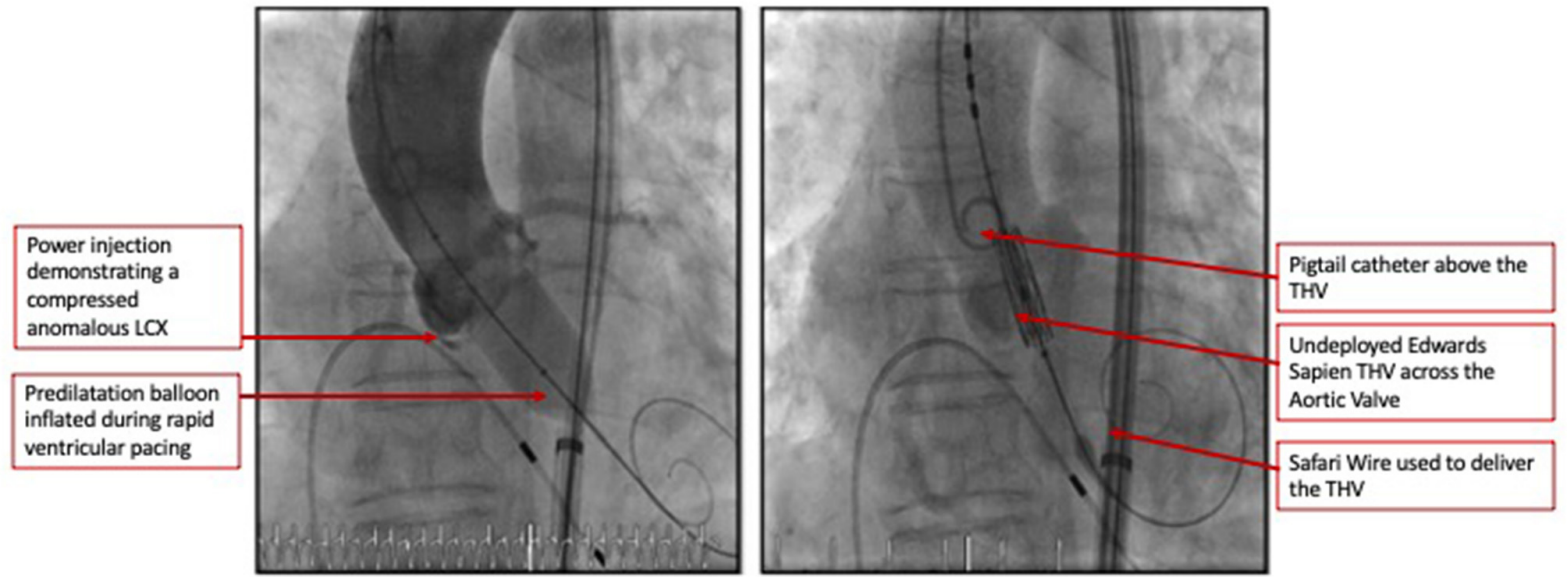
Coronary angiogram demonstrated a patent RCA and LM artery ostia with some compression of the LCX's mid segment. As such the decision was to proceed as planned with a high implant of the Edwards Sapien 3 valve.

**Figure 3:**
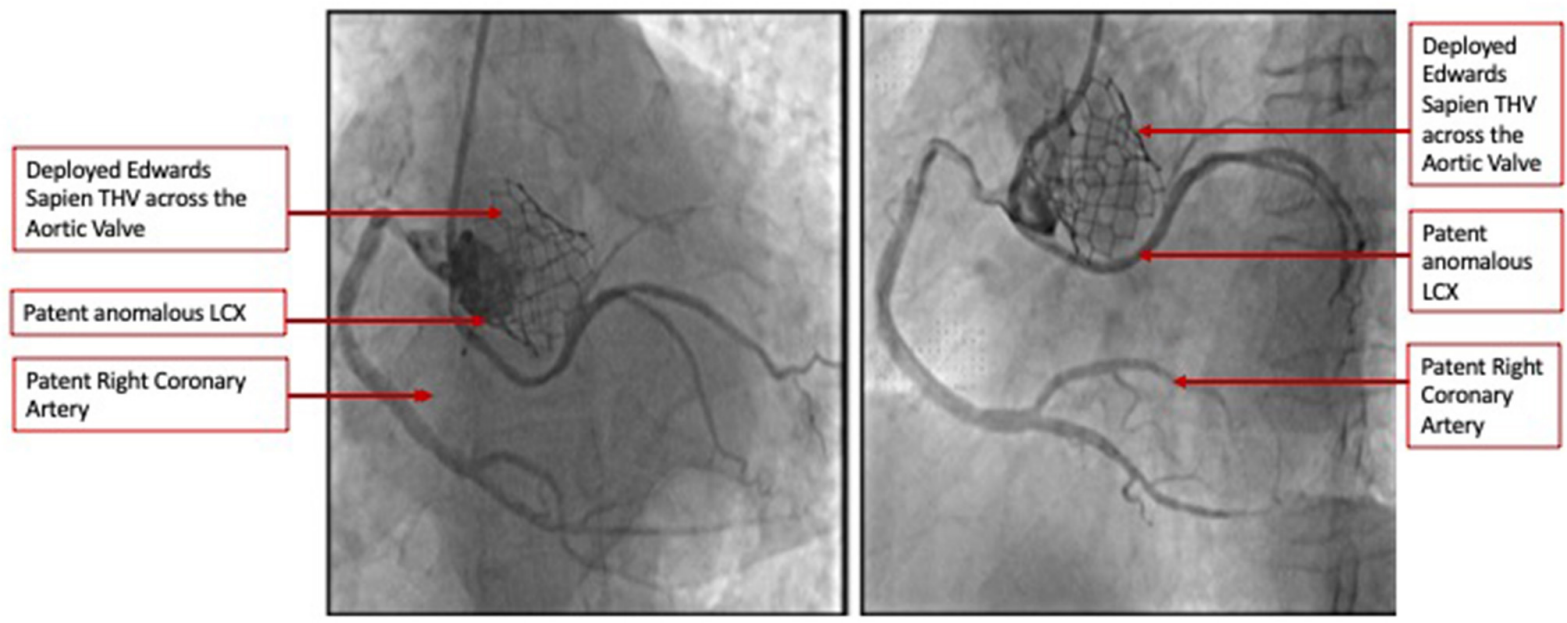
Selective coronary angiography demonstrating widely patent coronary arteries including the anomalous LCX in two orthogonal views.

**Figure 4:**
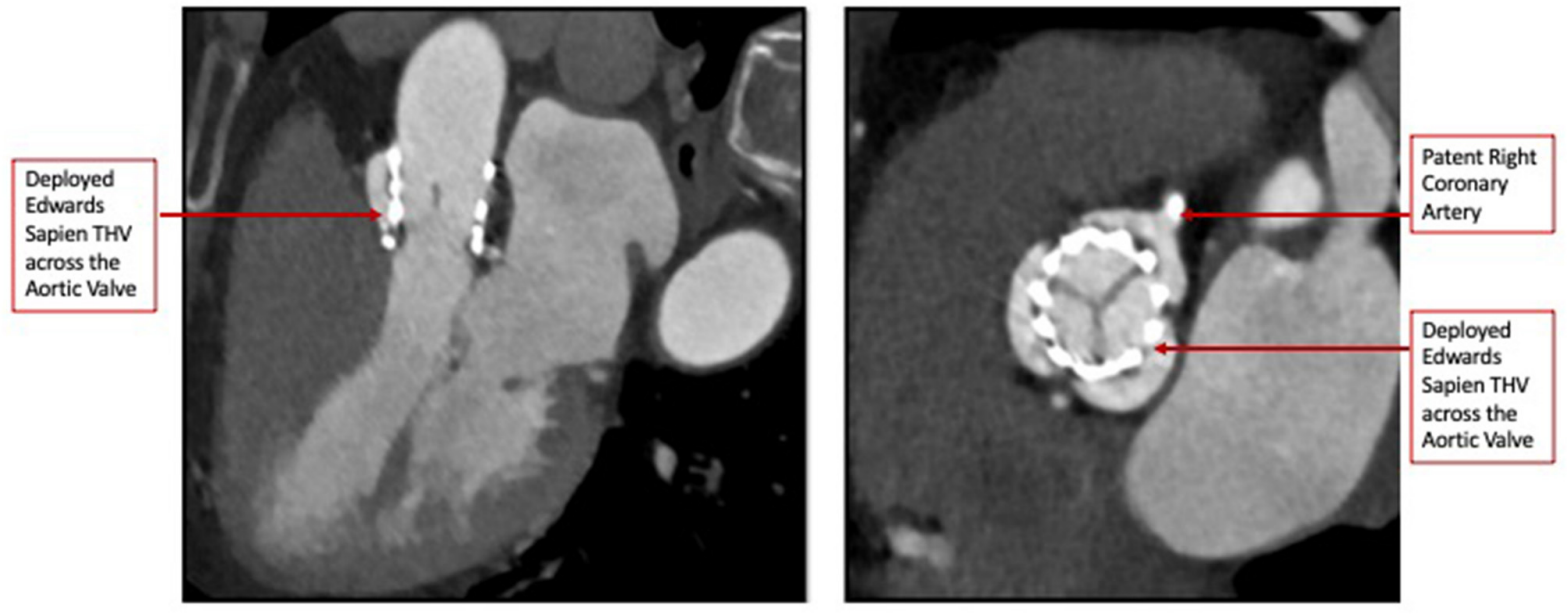
Post-TAVR Cardiac Computed Tomography images (Short and Long-axis) demonstrating thin mobile leaflets with no hypoattenuation.

The heart team-meeting consensus was to proceed with TAVR using an Edwards SAPIEN valve with high implant. Alternate access should be obtained to rescue the anomalous LCX. The patient underwent the procedure successfully as follows: Through the left common femoral vein sheath, a temporary pacing wire was inserted. Through the left arterial sheath, a pigtail was advanced. Through the right arterial sheath, an Edwards SAPIEN delivery sheath was advanced over a Sapphire wire that had been placed in the LV through the standard method. The valve was predilated using rapid pacing, and an angiogram through the pigtail catheter demonstrated a patent RCA, LCX, and LM artery ostial with some compression of the LCX midsegment. As such, the decision was to proceed as planned with a high implant of the Edwards SAPIEN valve (Figure 2). The implant was performed with rapid ventricular pacing. Angiography using a power injector confirmed appropriate expansion of the valve and no paravalvular regurgitation. Selective angiography of the LCX with a 6-French AL 0.75 guiding catheter confirmed widely patent coronary arteries (Figure 3). The echocardiogram further confirmed the angiographic findings. Post procedure, the gradient by echocardiography was 7 mmHg, and the EF had improved to 44.5% (Supplementary Figure 2). The improvement in EF could be due to inter-observer variability although it is uncommon to be this wide of a range. It may indicate timely replacement with improvement in the left ventricular systolic and diastolic functions. Imaging including cardiac MRI could identify fibrosis that may suggest poor recovery. This was not performed in this patient. The postoperative course was uneventful, and she was discharged on day 3 in stable condition without a need for a pacemaker on dual antiplatelet therapy (aspirin 81 mg and clopidogrel 75 mg daily). She was evaluated after 1 month at an outpatient clinic, where she was completely asymptomatic. A repeat CCT demonstrated thin leaflets with normal mobility and a widely patent LCX (Figure 4 and Supplementary Figure 3).

## Discussion

The role of CCT has conventionally been limited to the pre-procedure planning of TAVR. This case demonstrates the ability of CCT to accurately identify an anomalous LCX arising from the right coronary cusp, define its course between the aorta and left atrium, and provide accurate measurements of the distance of this vessel from the annulus at its origin and track it along its course. We were able to preemptively determine that the closest point to the annulus was at the proximal segment of the vessel. We were also able to determine the need for a high 90:10 implant to avoid vessel compression. Obstruction of anomalous vessels has been reported, and it is critical to identify and strategize appropriately to prevent such complications (6). Finally, we selected a balloon-expandable valve which has a more controlled and predictable deployment. The retrievable valve systems such as the Boston Scientific Lotus are not currently available in our region. The Boston Scientific ACURATE neo valve has an open-cell design and a lower metal-to-artery ratio than the Medtronic CoreValve. Both valves permit reasonable coronary access with commissural alignment; however, studies including that published by Tang et al. demonstrated a rate of right coronary artery overlap of 7.1% for both the CoreValve and ACURATE neo and no difference for the Edwards SAPIEN (7). We currently do not have data for anomalous vessel overlap. As such, we opted for the balloon-expandable Edwards SAPIEN valve. The limitation of CCT, however, was that the best annular projection could not identify the point at which the anomalous LCX traverses the annulus and hence would not permit us to use this particular angle to ensure a safe distance during deployment. As such, during the preparation for the procedure, we determined the need to selectively engage the LCX and maintain a wire in the event coronary compression occurs and requires intervention particularly after the predilatation was performed (8). Integrated or fusion imaging would be a useful technology for such cases (9). Since we did not have it available, it was crucial that all available imaging information was used including fluoroscopy and not limited to CCT. It is important to recognize that compression is a visual estimate at this time by both fluoroscopy and CCT with more objective quantification not being validated in such procedures.

It is important to recognize that the role of CCT was not restricted to the pre-procedure workup, but in the follow-up as well. We were able to non-invasively evaluate the valve position, leaflet thickness, and mobility and reassess the LCX patency in the follow-up CCT (10).

## Conclusions

TAVR is considered a minimally invasive interventional procedure with a high success rate largely due to meticulous pre-procedure planning and patient selection. This case demonstrates the role of CCT in mapping the course of anomalous LCX and defining the distance of the vessel to the AV annulus during the pre-procedure assessment. It also underscores the importance of CCT in the post-procedure evaluation of the valve itself and its relationship to the anomalous vessel.

## Data Availability Statement

The original contributions presented in the study are included in the article/Supplementary Material, further inquiries can be directed to the corresponding author/s.

## Ethics Statement

Written informed consent was obtained from the individual(s) for the publication of any potentially identifiable images or data included in this article.

## Author Contributions

All authors listed have made a substantial, direct and intellectual contribution to the work, and approved it for publication.

## Conflict of Interest

The authors declare that the research was conducted in the absence of any commercial or financial relationships that could be construed as a potential conflict of interest.

## Publisher's Note

All claims expressed in this article are solely those of the authors and do not necessarily represent those of their affiliated organizations, or those of the publisher, the editors and the reviewers. Any product that may be evaluated in this article, or claim that may be made by its manufacturer, is not guaranteed or endorsed by the publisher.
