# Supplementary figures and images for: Case Report: Transcatheter Aortic Valve Replacement in a Patient With Severe Aortic Stenosis, Left Ventricular Dysfunction, and an Anomalous Left Circumflex Artery
Source: Front Cardiovasc Med. 2021 Aug 18;8:721363. doi: 10.3389/fcvm.2021.721363 (PMC8416351; doi:10.3389/fcvm.2021.721363)

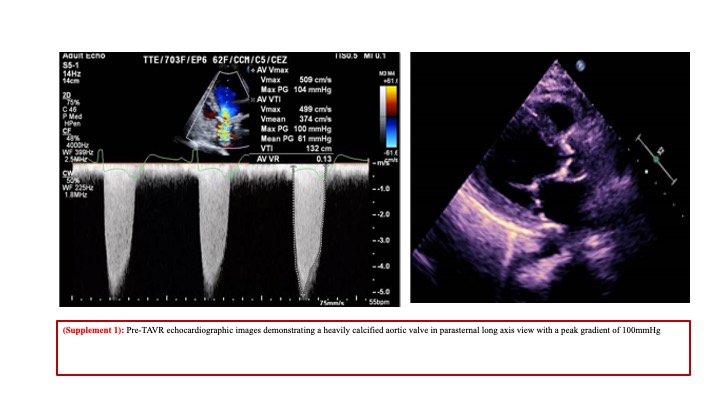

Supplement: Supplementary file 1 [file Image_1.jpeg]

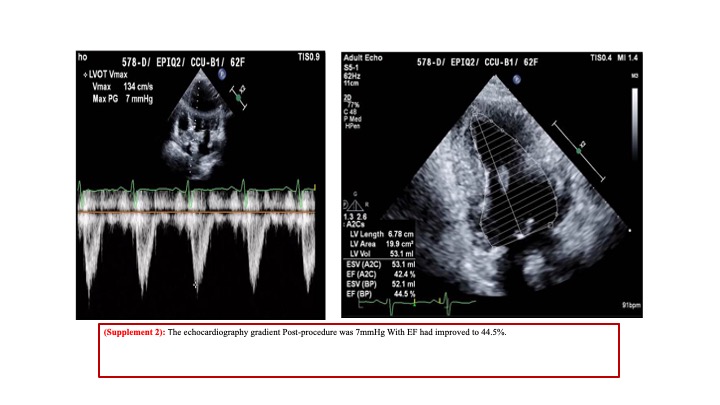

Supplement: Supplementary file 2 [file Image_2.jpeg]

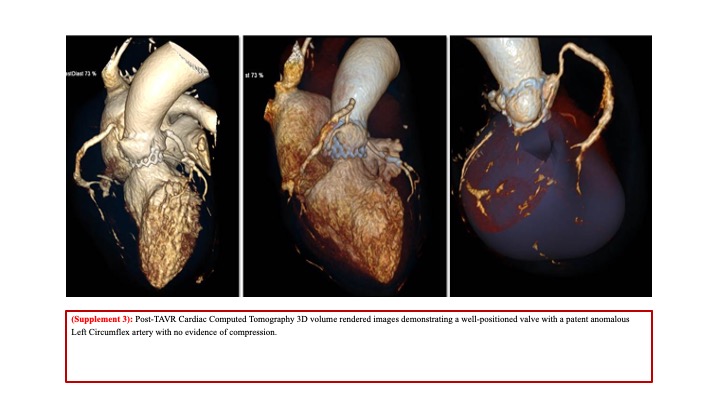

Supplement: Supplementary file 3 [file Image_3.jpeg]
